# Editorial: Stem cells and progenitor cells in ischemic stroke—fashion or future?

**DOI:** 10.3389/fncel.2015.00334

**Published:** 2015-08-25

**Authors:** Thorsten R. Doeppner, Dirk M. Hermann

**Affiliations:** Department of Neurology, University of Duisburg-Essen Medical SchoolEssen, Germany

**Keywords:** stem cells, cell transplantation, stroke, cerebral ischemia, neurogenesis, neuroregeneration

Despite recent achievements in re-canalizing stroke therapies (Campbell et al., 2015; Goyal et al., 2015), which ensure reduction of deficits in a significant patient faction due to combined systemic thrombolysis and interventional clot removal, a major need remains for restorative therapies in patients suffering from persistent neurological deficits despite optimized treatment. Although neurogenesis persists in the adult mammalian brain within distinct niches, such as the subventricular zone (SVZ) hosting stem cells and neural progenitor cells (NPCs) alike, the restorative potential of endogenous neurogenesis is generally insufficient and thus unable to support a full recovery of lost functions following stroke. Consequently, transplantation of exogenous NPCs and various other stem cell sources has emerged as a potential stroke treatment. Although adult NPCs are not integrated into residing neural networks, solid experimental data demonstrates beneficial effects in pre-clinical stroke models (Bacigaluppi et al., 2009; Doeppner et al., 2012). Thorough insights into stem cell actions have been obtained in experimental studies in recent years, which raise the questions about the clinical potential of stem cell-based therapies.

The mechanisms underlying post-stroke neurogenesis are diverse and highly complex, involving interactions of stem cells with extracellular matrix (ECM) constituents, microvascular cells, brain parenchymal cells, and immune cells, as summarized within this research topic by Hermann et al. (2014). The pivotal role of calpains, which are activated upon post-ischemic cellular calcium influx and control ECM remodeling, in post-stroke neurogenesis was now further analyzed by Machado et al. (2015) who provide compelling evidence that deletion of the endogenous calpain inhibitor calpastatin hampers the proliferation and migration of NPCs, whereas calpain inhibition increases NPC proliferation, migration speed, and migration distance. Accordingly, the modulation of calpains might be a potent tool to boost post-stroke neurogenesis. Representing a molecular substrate of calpains, the multifunctional ECM glycoprotein tenascin-C exemplifies in a particularly multi-faceted way how the characteristics of stem cells are modified by ECM constituents upon brain injury, as outlined by Roll and Faissner (2014). Casting new light onto the role of the cerebral microvasculature for post-stroke neurogenesis, Adamczak et al. (2014) provide a detailed non-invasive analysis of the dynamics of VEGF and its receptor VEGFR2 in a mouse model of focal cerebral ischemia. The authors describe active VEGFR2 signaling for as long as 2 weeks post-stroke that is likely to promote NPC migration and proliferation. Non-invasive imaging will greatly facilitate research on neurogenesis in the near future, as stressed by Aswendt et al. (2014), who systematically reviewed the requirements, advantages, and limitations of optical imaging as compared with existing imaging techniques.

In light of the insufficient neurorestorative capacity of endogenous neurogenesis, various studies aimed to support neurogenesis by cell transplantation. Due to their low immunogenicity and easy accessibility, mesenchymal stem cells (MSCs) were by far most often used in experimental stroke studies followed by NPCs. Yet, recovery-promoting actions can be achieved by various cell sources, as shown in the present research topic for amniotic fluid-derived stem cells, which protect the brain against ischemic injury (Tajiri et al., 2014). Yet, several open questions and pitfalls still have to be overcome to enable the translation of stem cell therapy from bench to bedside. These include the differentiation, fate and safety of transplanted cells as well as the contamination of grafted cells with feeder cells that could also pose a significant hazard to the recipient, as described by Ikegame et al. (2014) and Molcanyi et al. (2014). As neither endogenous nor exogenous adult stem cells are integrated into residing neural networks, it stands to reason whether or not transplantation of stem cells is mandatory for induction of neuroprotection. Indeed, recent evidence suggests that extracellular vesicles (e.g., exosomes) containing non-coding RNAs might be the biologically active mediator of stem cell-induced neuroprotection and brain plasticity (Xin et al., 2014). Extracellular vesicles might allow for evading cell-based safety issues. This concept deserves further in-depth evaluation in experimental systems and might offer itself for subsequent proof-of-concept studies in human stroke patients.

The vast majority of pre-clinical stroke studies were hitherto limited to adolescent, otherwise healthy rodents, which poorly reflects the clinical situation of elderly, multimorbid stroke patients. In order to analyze consequences of arterial hypertension, a particularly prevalent stroke risk factor, for responses to stem cell therapy, Diederich et al. (2014) evaluated effects of granulocyte-colony stimulating factor (G-CSF) and bone marrow derived mononuclear cells (BM-MNCs) in spontaneously hypertensive rats exposed to stroke (Diederich et al., 2014). In their study, the combined delivery of G-CSF and BM-MNCs was not superior to G-CSF alone. Most importantly, single treatment with BM-MNCs did not yield any therapeutic effect, in line with earlier data from this group (Minnerup et al., 2014). The evaluation of risk factors, such as arterial hypertension, will require intensified research in the future. Beside co-morbidities, age-related changes of the cerebral microenvironment have a strong impact on post-stroke brain remodeling according to Popa-Wagner et al. (2014) who claim that the aged brain is not refractory to post-stroke plasticity after cell grafting. Yet, significant age-related changes have been identified, i.e., a higher vulnerability to ischemic insults, a reduced rate of neurogenesis, and a delayed initiation of neurological recovery, which should carefully be considered in the implementation of clinical proof-of-concept studies. In view of age-related specificities, the neonatal mammalian brain might provide a particularly suitable environment for cell transplantation studies according to van Velthoven et al. (2014).

In the context of cell therapy, the selection of behavioral tests has repeatedly been criticized. Thus, it was objected that behavioral test batteries are optimized to detect functional neurological improvements, the significance of which under clinical conditions remains obscure. In a systematic study including as many as 12 motor-coordination and cognitive tests, Doeppner et al. (2014) now refute these criticisms, demonstrating consistent improvement of neurological function in response to NPC delivery across a large variety of tests. These data provide strong evidence regarding the potency of stem cells in experimental stroke settings, supporting the contributors' overall view that cell-based therapies have true potential for clinical translation. In light of a plethora of pre-clinical studies demonstrating successful post-stroke neurological recovery and brain remodeling after stem cell transplantation, first clinical trials have already been performed on small patient cohorts, as summarized by Doeppner and Hermann (2014). Promising data were obtained particularly following intravenous MSC delivery that until now, however, lack appropriate control groups. Stringent proof-of-concept strategies including clear-defined goals, measures, and actions will now have to be implemented, which further bridge the gap between the laboratory bench and the clinical setting. With such information, controlled clinical proof-of-concept studies may then be scheduled providing ultimate proofs whether cell-based therapies are able to enhance neurological recovery post-stroke.

## Conflict of interest statement

The authors declare that the research was conducted in the absence of any commercial or financial relationships that could be construed as a potential conflict of interest.
